# Supplementary material for: Monitoring of language selection errors in switching: Not all about conflict
Source: PLoS One. 2018 Nov 26;13(11):e0200397. doi: 10.1371/journal.pone.0200397 (PMC6261013; doi:10.1371/journal.pone.0200397)
Supplement: S1 Appendix — A full list of cognate and noncognate words used in the study. (DOCX) [file pone.0200397.s001.docx]

# S1 Appendix A: Materials

|  | Noncognates | | Cognates | |
| --- | --- | --- | --- | --- |
|  | English name | Dutch name | English name | Dutch name |
| 1 | basket^*^ | mand | bee^*^ | bij |
| 2 | box | doos | beard | baard |
| 3 | butcher^*^ | slager | bread | brood |
| 4 | button | knoop | bridge | brug |
| 5 | cage^*^ | kooi | cow | koe |
| 6 | corn | mais | butter^*^ | boter |
| 7 | flower | bloem | hat | hoed |
| 8 | frog | kikker | king | koning |
| 9 | girl | meisje | magnet | magneet |
| 10 | horse | paard | mask | masker |
| 11 | key | sleutel | moon | maan |
| 12 | knife | mes | needle | naald |
| 13 | map | kaart | paper | papier |
| 14 | mirror | spiegel | pumpkin | pompoen |
| 15 | mountain | berg | shell | schelp |
| 16 | pillow^*^ | kussen | shoe | schoen |
| 17 | shark | haai | soldier | soldaat |
| 18 | shower | douche | star | ster |
| 19 | tail | staart | table | tafel |
| 20 | tree | boom | tiger | tijger |

^*^ These pictures were drawn from scratch.
