# Supplementary material for: Monitoring of language selection errors in switching: Not all about conflict
Source: PLoS One. 2018 Nov 26;13(11):e0200397. doi: 10.1371/journal.pone.0200397 (PMC6261013; doi:10.1371/journal.pone.0200397)
Supplement: S2 Appendix — All the categories and the percentages of each type of speech errors coded in the study. (DOCX) [file pone.0200397.s002.docx]

# S1 Appendix B: Error coding

|  |  | Description | Percentage (%) | |
| --- | --- | --- | --- | --- |
| Language selection errors | 1 | wrong language, correct word (complete response) | 16.4 |  |
| Other errors | 2 | wrong word, correct language (complete response) | 0.4 |  |
|  | 3 | wrong language, wrong word (complete response) | 0.1 |  |
|  | 4 | self-correction, wrong language | 1.4 |  |
|  | 5 | self-correction, wrong word | 0.2 |  |
|  | 6 | disfluency/self-correction for cognates (e.g., b-bread) | 0.2 |  |
|  | 7 | fail to respond | 0.2 |  |
|  | 8 | incomplete response, wrong language | 0.2 |  |
|  | 9 | incomplete response, wrong word | 0.1 |  |
|  | 10 | dysfluency, correct response | 0.6 |  |
|  | 11 | dysfluency, incorrect response | 0.2 |  |
|  | 12 | errors on nonoptimal item names (mandje, knoopje) | 0.0 |  |
|  | 13 | multicategory, uncategorized | 1.3 |  |
|  | 14 | RT outliers in correct responses | 1.1 |  |

*Note*. Interlingual errors: 1, 3, 6, 7, 8, 11, 13.
