# Supplementary material for: Monitoring of language selection errors in switching: Not all about conflict
Source: PLoS One. 2018 Nov 26;13(11):e0200397. doi: 10.1371/journal.pone.0200397 (PMC6261013; doi:10.1371/journal.pone.0200397)
Supplement: S3 Appendix — The linear mixed effect models used for analyses in the study. (DOCX) [file pone.0200397.s003.docx]

# S1 Appendix C: Linear mixed effect models

## Analysis of Switch Trials

### *Language selection errors*

glmer.LSerror.switch =

glmer(LSError~Lang + (1+Lang|pNum) + (1+Lang|PicNam),data=mydata.4ER.switch, family="binomial", control = glmerControl(optimizer = "bobyqa"))

### *RTs*

glmer.RT.switch =

glmer(RT~Lang + (1+Lang|pNum) + (1+Lang|PicNam),data=mydata.4RT.switch, family = Gamma(link = "identity"), control=glmerControl(optimizer = 'bobyqa'))

## Analysis of Repeat Trials

### *Language selection errors*

glmer.LSerror.repeat =

glmer(LSError ~ Lang*CogSta + (1+Lang*CogSta|pNum) + (1+Lang+CogSta|PicNam), data=mydata.4ER.repeat, family="binomial", control = glmerControl(optimizer = "bobyqa"))

# using Lang*CogSta|PicNam does not converge

### *RTs*

glmer.RT.repeat =

glmer(RT ~Lang*CogSta + (1+Lang*CogSta|pNum) + (1+Lang*CogSta|PicNam), data=mydata.4RT.repeat, family = Gamma(link = "identity"), control=glmerControl(optimizer = 'bobyqa'))

# IF there is an interaction

glm.RT.repeat.L1 = glmer(RT ~CogSta + (1+CogSta|pNum) + (1+CogSta|PicNam),

data=mydata.4RT.repeat[mydata.4RT.repeat$Lang=="Dutch",], family = Gamma(link = "identity"), control=glmerControl(optimizer = 'bobyqa'))

glm.RT.repeat.L2 = glmer(RT~CogSta + (1+CogSta|pNum) + (1+CogSta|PicNam),

data=mydata.4RT.repeat[mydata.4RT.repeat$Lang=="English",], family = Gamma(link = "identity"), control=glmerControl(optimizer = 'bobyqa'))
